# The Book World of Medicine and Science

**Published:** 1911-11-25

**Authors:** 


### The Book World of Medicine and Science.

Voice and Its Natural Development. By Herbert Jennings. Illustrated by photographs and drawings. Pp. 220. Crown 8vo. (London: George Allen and Co., Ltd. 1911. Price 3s. 6d. net.)

It is probably no exaggeration to say that the English as a nation do not enunciate clearly; the average Englishman's delivery is too often loose and muffled, and has been truthfully described as "fluffy." From the lips of educated foreigners our language is often heard to advantage, because they make better use of the vowels to enrich the language and enable the consonants to be pronounced without harshness. The author of this book has a great deal to say about the defects not only of our speech but also of our carriage and of our breathing and the misuse of our larynges, all of which he proposes to remedy by a certain system of vocal development of his own. Incidentally other systems seem to be full of faults since their results are, according to the author, very deplorable. However, Mr. Jennings has also a great deal of sound advice to give on physical training and breath control, which are naturally of fundamental importance in the development of the voice. Numerous exercises are given on articulation, tone cultivation, and emphasis. On the subjects of deportment and gesture and public speaking some useful advice is to be found. The book suffers, especially in the first half, from its verbose style, but the subject is undoubtedly a difficult one to deal with in silent print. The observance of the principles advocated and the practice of the physical exercises will certainly do any one good—possibly they would even make a man of the slouching school-boy who forms the subject of one of the illustrations.

